# Supplementary figures and images for: A multispecies framework for modeling adaptive immunity and immunotherapy in cancer
Source: PLoS Comput Biol. 2023 Apr 21;19(4):e1010976. doi: 10.1371/journal.pcbi.1010976 (PMC10155959; doi:10.1371/journal.pcbi.1010976)

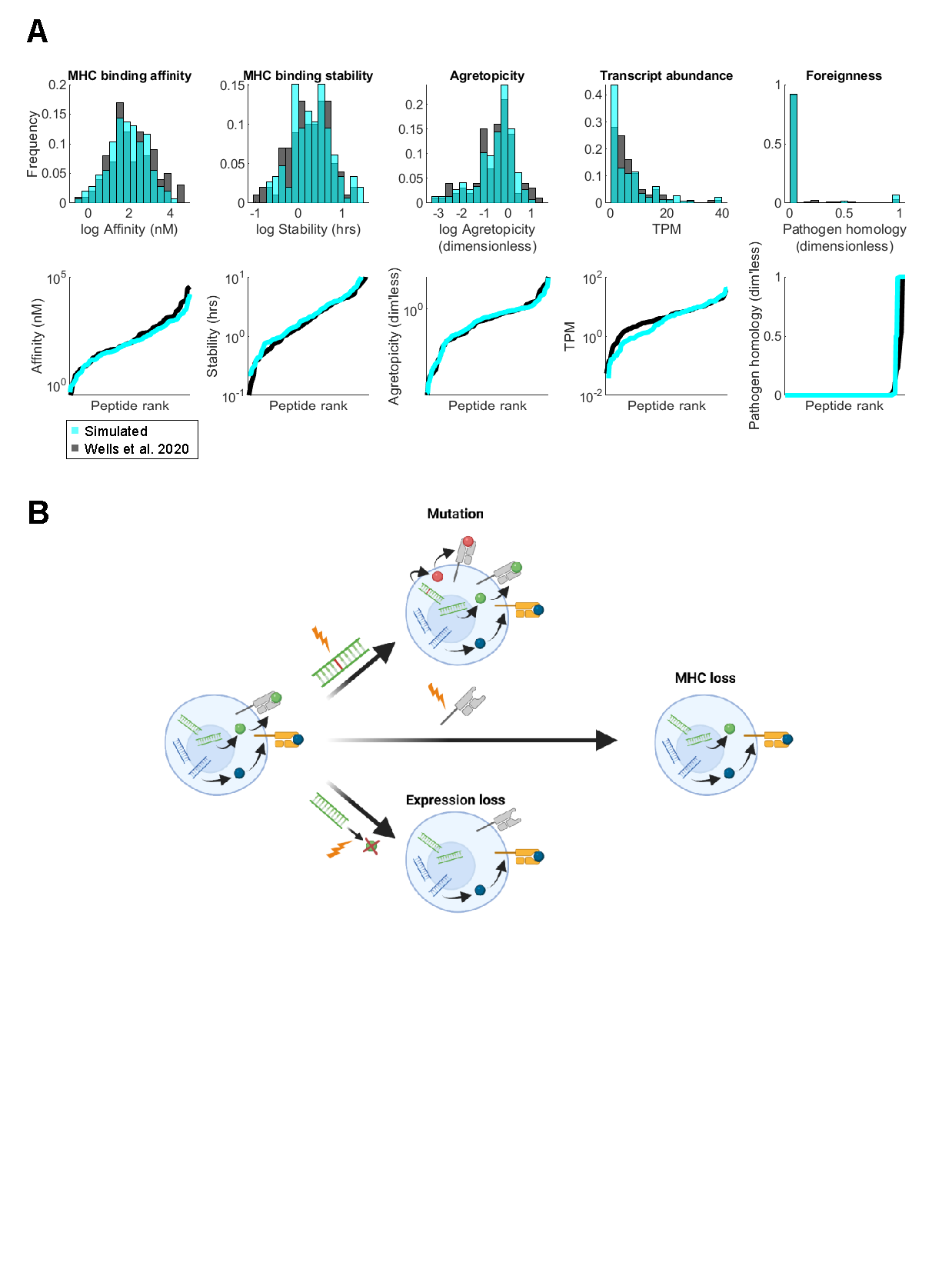

Supplement: S1 Fig — A. Each neoantigen was assigned values for the TESLA parameters drawn from the distributions for NSCLC tumors in [4]. TPM, transcripts per million. Distributions (top) and values (bottom) of sampled and experimental parameters from 146 NSCLC-derived peptides are shown. B. Schematic of stochastic neoantigen events (created with BioRender.com). A founder cell was established with a random number of neoantigens and assigned a neoantigen gain rate from a lognormal distribution. Each neoantigen was also assigned to be presented by one of three MHC alleles. Neoantigen and MHC expression could also be lost by a cell during tumor growth. (TIF) [file pcbi.1010976.s001.tif]

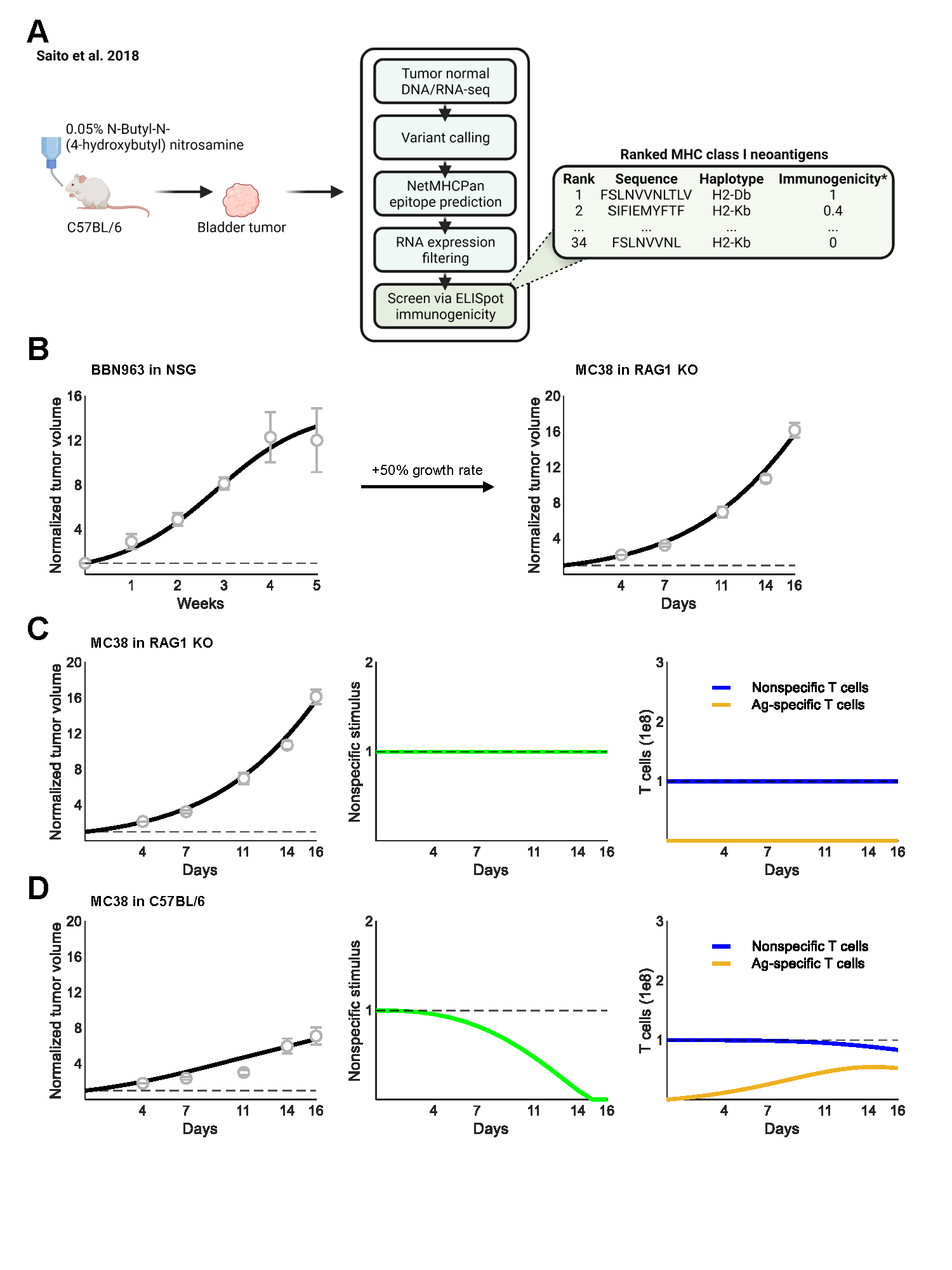

Supplement: S2 Fig — A. Schematic of BBN963 cells establishment by chronic exposure of C57BL/6 mice to 0.05% N-Butyl-N-(4-hydroxybutyl) nitrosamine (created with BioRender.com). Neoantigens were called and screened for bona fide immunogenicity in an ex vivo ELISpot assay. Additional details can be found in [24]. B. Growth dynamics of MC38 were simulated using the same parameters as BBN963 cells, except growth rate, which was increased 50% [26]. C. Simulated tumor volumes (left, black) normalized to baseline volume (dashed gray line) compared against growth dynamics in RAG1 KO mice reported in [26] (gray circles and error bars). Nonspecific stimulus (center) and CTL populations (right) are shown. Dashed lines, 1. D. Simulated tumor volumes (left, black) normalized to baseline volume (dashed gray line) compared against growth dynamics in C57BL/6 mice reported in [26] (gray circles and error bars). Nonspecific stimulus (center) and CTL populations (right) are shown. Dashed lines, 1. (TIF) [file pcbi.1010976.s002.tif]

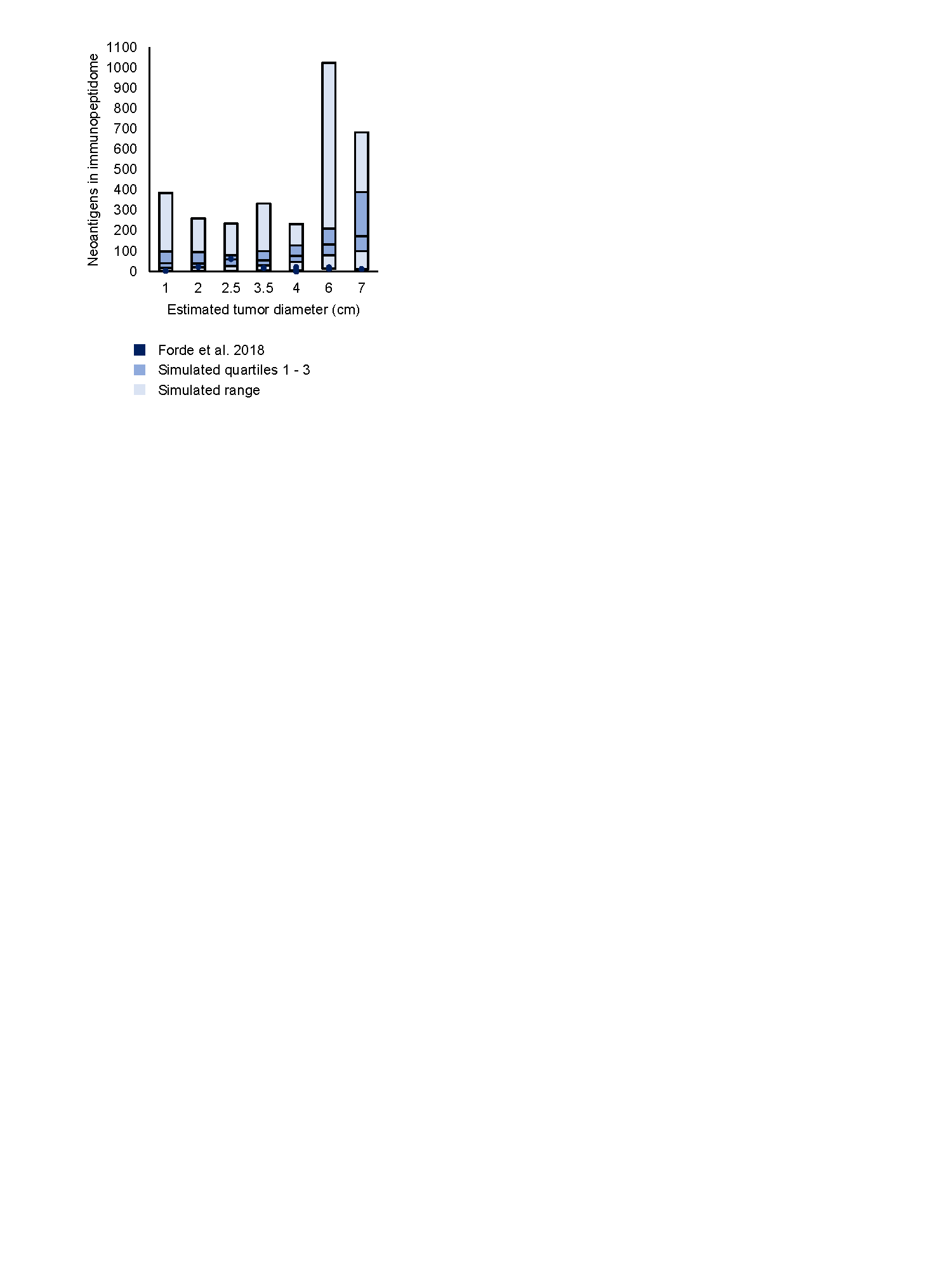

Supplement: S3 Fig — A. Neoantigen predictions reported in [27] of 12 patients with sufficient pretreatment tissue to characterize candidate neoantigens were assessed. Total neoantigens were calculated by filtering positive netCTL classification. To apply Wells’ criteria [4], we calculated agretopicity by taking the ratio of somatic and wild-type MHC binding affinity. For predictions containing somatic peptides of identical sequences but different HLA alleles, the stronger predicted binding was used. Binding stabilities were calculated with NetMHCStabPan using default parameters. As biopsies were from a single region, neoantigen clonality was not assessed. Tumor diameters were estimated for each tumor using conventional NSCLC staging criteria. For each of seven discrete estimated tumor diameters, a cohort of 40 tumors was simulated. (TIF) [file pcbi.1010976.s003.tif]

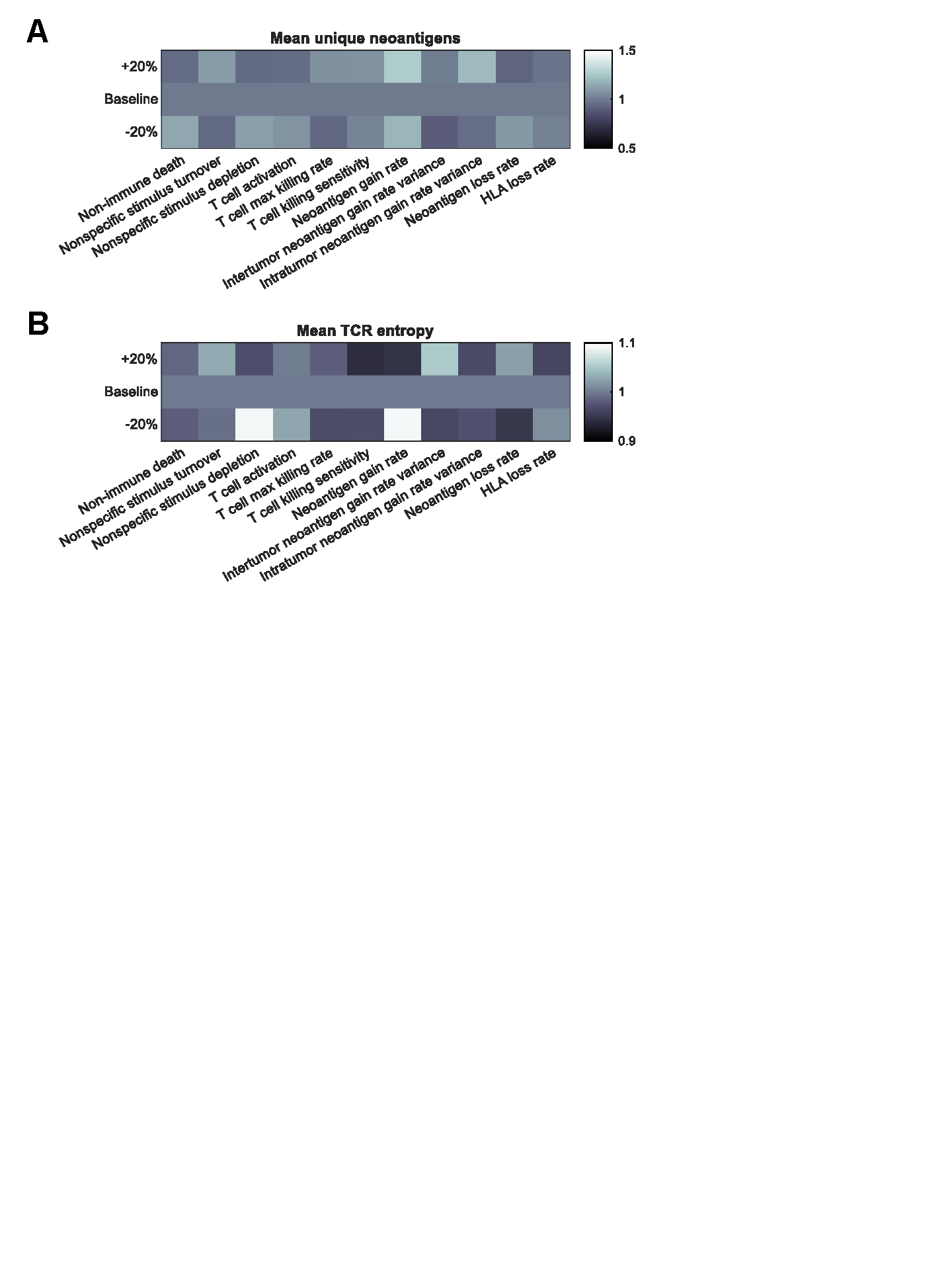

Supplement: S4 Fig — A. Mean number of unique neoantigens relative to baseline (N = 100 simulations per condition). No significant fold differences in neoantigen number were found from pairwise Student’s t-tests with nominal and Bonferroni-corrected significance thresholds. B. Mean TCR entropy relative to baseline (N = 100 simulations per condition). No significant fold differences in Shannon entropy were found from pairwise Student’s t-tests with nominal and Bonferroni-corrected significance thresholds. (TIF) [file pcbi.1010976.s004.tif]
